# Anti-NMDA-receptor encephalitis and MOGAD associated optic neuritis: a case series

**DOI:** 10.3389/fneur.2026.1869098

**Published:** 2026-07-10

**Authors:** Pooja Parthasarathi, Michael Dattilo, Jason Peragallo

**Affiliations:** Department of Ophthalmology, Emory University, Atlanta, GA, United States

**Keywords:** encephalitis, MNOS, MOGAD, NMDA, optic neuritis

## Abstract

**Introduction:**

Anti-N-methyl-D-aspartate receptor (NMDAR) encephalitis is a well-recognized autoimmune condition that often presents with neuropsychiatric symptoms and seizures. Myelin oligodendrocyte glycoprotein antibody-associated disease (MOGAD) often manifests as optic neuritis and, less frequently, as acute demyelinating encephalomyelitis or transverse myelitis. The co-occurrence of anti-NMDAR encephalitis and MOGAD is becoming increasingly recognized, but clinical series remain limited.

**Description of cases:**

We present three patients with anti-NMDAR encephalitis and MOGAD optic neuritis (ON): two men, aged 19 and 26, and one woman, aged 36. Clinical presentations, signs, investigations, and management of each case are discussed. The 26-year-old man presented with altered mental status and concurrent vision loss. The 36-year-old woman presented with altered mental status during the encephalitis episode and developed vision loss 4 months after encephalitis. The 19-year-old man with a prior history of altered mental status, diagnosed with NMDA encephalitis 9 years earlier, presented with headache and vision loss. Abnormal T2/FLAIR lesions in the brain and/or spinal cord during the encephalitis episode and unilateral or bilateral optic nerve enhancement during the optic neuritis episode were detected on brain and orbital magnetic resonance imaging (MRI) in all patients. All patients tested positive for cerebrospinal fluid (CSF) anti-NMDAR antibodies during the encephalitis episode and had positive serum MOG titers during the optic neuritis episode. Each patient presented with bilateral, asymmetrically reduced visual acuity and diminished color vision. One patient exhibited bilateral temporal optic nerve pallor, while two patients had bilateral optic nerve edema. The diagnostic work-up revealed positive serum MOG titers (1:100, 1:10,000, and 1:10,000 in the 36/F, 26/M, and 19/M, respectively). The 36-year-old woman was treated with intravenous (IV) steroids, plasma exchange (PLEX), and rituximab during the encephalitis episode. During the optic neuritis episode, she was treated with IV steroids, IV immunoglobulin (IVIG), and rituximab, followed by long-term rituximab maintenance therapy. The 19-year-old man was treated for encephalitis with IV steroids, IVIG, and rituximab. During his optic neuritis episode, he received IV steroids, IVIG, and tocilizumab, followed by long-term tocilizumab maintenance therapy. The 26-year-old man was treated with IV steroids, PLEX, and rituximab during the acute episode, followed by long-term IVIG maintenance therapy. After achieving 2 years of stability that prompted the discontinuation of IVIG, the patient experienced a MOG-IgG-positive relapse 5 months later. This relapse was marked by a seizure-like episode and the appearance of new lesions on MRI. The acute symptoms resolved after treatment with intravenous steroids and IVIG. Subsequently, the patient was initiated on an indefinite maintenance IVIG regimen. Visual acuity in all patients improved to their baseline levels following treatment.

**Conclusion:**

This series highlights the emerging overlap between anti-NMDAR encephalitis and MOGAD optic neuritis. Optic neuritis may occur months to years after encephalitis, underscoring the need for careful monitoring of patients with anti-NMDAR encephalitis who develop new visual symptoms. Dual autoimmunity may represent a distinct phenotype with implications for long-term immunotherapy.

## Introduction

Anti-N-methyl-D-aspartate receptor (NMDAR) encephalitis is a well-recognized autoimmune condition caused by cerebrospinal fluid (CSF) antibodies against the NMDA receptor in the central nervous system (CNS) (1). NMDA receptors are ligand-gated calcium channels that mediate synaptic plasticity, learning, memory, and neuronal development (2). Anti-NMDAR encephalitis was first described in a 2005 case series involving four women with ovarian teratoma who developed neuropsychiatric manifestations (3). The patients’ serum or CSF showed immunolabeling of antigens expressed at the cytoplasmic membrane of hippocampal neurons that were later identified to be NMDA receptors (4). Anti-NMDAR encephalitis is characterized by a 1–2 week period of predominantly psychiatric symptoms, such as delusions, hallucinations, mania, agitation, disorganized thinking, changes in speech, catatonia, and insomnia at onset, followed by neurological symptoms, such as movement abnormalities, seizures, dysautonomia, and hypoventilation in the weeks to months that follow. Following the acute phase, patients often experience prolonged deficits lasting for years, which may include executive dysfunction, impulsivity, disinhibition, and sleep abnormalities (5). A recent population-based study has estimated the incidence per million person-years to vary by race, with the lowest incidence in Caucasians (0.4), followed by Asians (2.02), Hispanics (2.17), and Africans (2.94). The median age of onset was 23.7 years, with women constituting 64% of the cases (6).

Myelin oligodendrocyte glycoprotein antibody-associated disease (MOGAD) most frequently manifests as optic neuritis (ON) caused by CSF antibodies against Myelin oligodendrocyte glycoprotein (MOG). MOG proteins are minor myelin components located on the outermost surface of myelin. Although their exact function remains unknown, they serve as a marker of oligodendrocyte maturity and are exclusively expressed in the CNS (7). Anti-MOG antibodies were first described in 2007 in pediatric patients with demyelinating encephalitis (8). In 2011, they were identified in adult patients with ON using cell-based assays (9). MOGAD-ON typically presents as acute, painful, severe vision loss (bilateral in up to 50% patients), and 70–80% of patients demonstrate optic nerve edema with optic nerve or perineural enhancement on orbital magnetic resonance imaging (MRI) (10). A recent population-based study in the US estimated the incidence of MOGAD to be 3.00 cases per million person-years in Olmsted County and 1.18 cases per million person-years in Martinique, with a median age of onset of 27 years and 38% of the affected individuals being women (11).

The co-existence of MOGAD-ON and anti-NMDAR encephalitis, known as MOG-Ab and NMDA-Ab overlapping syndrome (MNOS), was first reported in 2014 (12). Epitope spreading is a proposed mechanism to explain this co-occurrence. According to this theory, initial autoimmune-mediated CNS tissue damage releases sequestered antigens that can trigger a secondary immune response against additional CNS targets (13).

Approximately 3–5% of patients with anti-NMDAR encephalitis develop clinical or radiological signs of a demyelinating disorder (5). A recent systematic review analyzed 25 studies and identified 256 patients with anti-NMDAR encephalitis and co-existing demyelinating syndromes. Among these 256 patients, 94.5% had MOGAD, 4.7% had neuromyelitis optica spectrum disorder (NMOSD), and 0.8% had multiple sclerosis. However, it is important to note that this review included studies using a range of fixed and live cell-based assays, different cutoff titers, and studies that were conducted prior to the 2023 International MOGAD diagnostic criteria. Optic nerve involvement was noted in 25% of these patients. Additionally, temporal lobe involvement was observed in 50%, frontal lobe involvement in 40%, spinal cord involvement in 30%, and basal ganglia involvement in 30% (14).

The co-occurrence of anti-NMDAR encephalitis and MOGAD occurs more frequently in men (15, 16). Patients with overlapping anti-NMDAR encephalitis and demyelinating syndromes tend to have poorer outcomes compared to those with isolated anti-NMDAR encephalitis. Another study compared 23 patients with MNOS, 51 patients with isolated anti-NMDA encephalitis, and 30 patients with isolated MOGAD. Compared to the anti-NMDAR encephalitis group, the MNOS group had a higher proportion of men and a higher rate of brainstem involvement on MRI. The MNOS group had fewer patients with psychiatric symptoms compared to the anti-NMDAR encephalitis group. The proportion of patients with psychiatric disorders, disturbances of consciousness, cognitive impairment, and sleep disorders in the MNOS group was higher than that in the MOGAD group. In contrast, the MNOS group had a lower proportion of patients with visual impairment and limb weakness (16).

To the best of our knowledge, sequential anti-NMDAR encephalitis followed by MOGAD-ON has been rarely reported in the literature (17, 18), while simultaneous anti-NMDAR encephalitis and MOGAD-ON has been documented more frequently (12, 19–21). We present two patients with sequential anti-NMDAR encephalitis followed by MOGAD-ON and one patient with simultaneous anti-NMDAR encephalitis and MOGAD–ON, all seen at a tertiary medical center between 2020 and 2026 (Supplementary Table 1).

## Case description

### Case 1

A 36-year-old woman consulted a neurologist due to new-onset headaches. Brain MRI, with and without contrast, was reportedly normal. A lumbar puncture (LP) showed an opening pressure of 36 cm H_2_O with neutrophilic lymphocytosis (87 WBCs). Acetazolamide was prescribed. Four days later, she developed an altered mental status and aphasia. Upon her presentation to the emergency department, CT/CTA of the head and repeat MRI of the brain were found to be normal. Continuous electroencephalography (cEEG) revealed frequent left-sided periodic discharges associated with left-sided cortical dysfunction, suggestive of severe generalized encephalopathy. Acetazolamide was discontinued. On ophthalmology examination, which was limited by altered mental status, she was found to have visual acuity of at least 20/50 in both eyes and no evidence of disc edema or optic neuropathy. A repeat LP showed an opening pressure of 18 cm H_2_O The CSF analysis showed 157 nucleated cells (normal 0–5/uL) (88% lymphocytes, normal 40–80%), protein level at 51 mg/dL (normal 15–45 mg/dL), and glucose level at 58 mg/dL (normal 40–70 mg/dL). She became febrile following an episode of aspiration; as a result, she was started on empiric IV acyclovir, vancomycin, and ceftazidime. A repeat brain MRI, performed with and without contrast, showed an increased T2/FLAIR signal in the left frontal, temporal, and parietal lobes, along with scattered areas of leptomeningeal enhancement (Figure 1).

**Figure 1:**
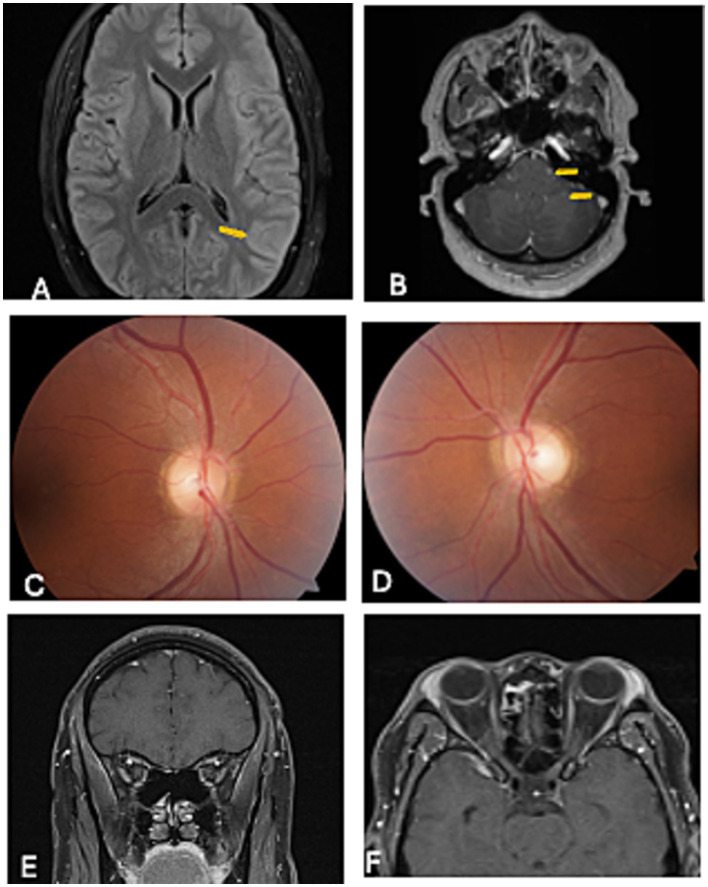
Case 1. **(A)** Axial MRI of the brain shows T2/FLAIR hyperintensity and gyriform edema of left frontal, temporal and parietal lobe demonstrated by yellow arrows; **(B)** axial MRI of the brain T1 post-contrast sequence shows punctate enhancing lesions of pons and cerebellum demonstrated by yellow arrows; **(C, D)** digital fundus photographs show temporal pallor of left eye; **(E)** left greater than right perineural enhancement on coronal orbital MRI T1 post-contrast fat saturated sequence; **(F)** left greater than right perineural enhancement on axial orbital MRI T1 post-contrast fat saturated sequence.

The CSF meningitis/encephalitis panel was negative. CSF cultures showed no growth. A repeat LP on day 10 of admission showed an opening pressure of 22 cm H_2_O, with 49 nucleated cells (94% lymphocytes), protein level at 21 mg/dL, and glucose level at 63 mg/dL. Repeat EEGs showed “Extreme delta brush,” which was highly suggestive of NMDA encephalitis. IV antibiotics were discontinued, and she was treated with IV Solumedrol for 5 days, but there was a poor clinical response. She received seven sessions of plasma exchange (PLEX). A repeat brain MRI on day 16 showed a partial treatment response with unchanged T2/FLAIR signal in the left frontal/temporal lobes and decreased leptomeningeal involvement. Rituximab was administered on days 22 and 36. On day 24, the results from the CSF autoimmune encephalopathy panel were positive for anti-NMDAR antibodies (titer 1:256), and serum NMDA titer was also positive (1:80). The patient developed acute respiratory failure, autonomic instability, and refractory seizures, which necessitated prolonged mechanical ventilation. She was transferred to an acute rehabilitation facility approximately 3 months post-admission and was discharged home 1 month later.

Four months after the initial admission (before her third dose of rituximab), she noticed blurry vision in her left eye (OS). An MRI of the brain and orbits, with and without contrast, showed that the previous leptomeningitis and encephalitis has resolved, with new perineural enhancement of the left optic nerve greater than that of the right at the optic canal (Figure 1). Neuro-ophthalmology consultation showed a visual acuity of 20/20 in the right eye (OD) and 20/400 in the left eye (OS), along with a left relative afferent pupillary defect (RAPD), dyschromatopsia OS, and temporal pallor of the left optic nerve (Figure 1). Optical coherence tomography (OCT) scans of the optic nerve head and macula showed peripapillary retinal nerve fiber layer (pRNFL) and ganglion cell complex (GCC) thinning in the left eye. An extensive laboratory evaluation of her left optic neuropathy was positive for serum MOG antibody (1:100). CSF MOG antibody levels were not obtained. She was admitted and treated with IV steroids (1 g per day) for 5 days, followed by 5 days of IV immunoglobulin (IG) treatments. Her visual acuity OS improved to 20/200, and she was discharged on oral prednisone.

At her neuro-ophthalmology follow-up 1 month later, her OS visual acuity had improved to 20/125, with stable pRNFL and GCC thickness on OCT. Rituximab maintenance was initiated. Follow-up brain and orbits MRI, with and without contrast, performed 3 months after her ON episode, showed interval resolution of her bilateral perineural enhancement. At her 5-month neuro-ophthalmology follow-up, her visual acuity had improved to 20/40 + 1 OS; she continued to have severe dyschromatopsia OS and temporal pallor of both optic nerves. OCT RNFL and GCC were thin but stable OS. She was maintained on rituximab, and, at her 1-year neuro-ophthalmology follow-up, her afferent visual function had further improved; visual acuity was 20/25 + 2 OS, and she had mild residual dyschromatopsia with stable OCT RNFL and GCC thickness OS.

### Case 2

A 19-year-old man with a significant history of anti-NMDAR encephalitis diagnosed 9 years earlier—characterized by altered mental, MRI at the time showing diffuse demyelination (Figure 2) and CSF positivity, requiring corticosteroids, IVIG, and rituximab maintenance therapy—presented with an acute onset of headache and significant blurred vision OD. Initial management at an outside emergency department included a migraine cocktail (IM Solumedrol, promethazine, ketorolac, and diphenhydramine), which improved his headache but not his vision. He subsequently developed persistent vomiting. Four days after onset, he experienced sudden blurry vision OS, and an optometric evaluation revealed bilateral optic disc edema. Neurology consultation at an outside hospital initially attributed findings to idiopathic intracranial hypertension (IIH) based on symptoms, despite a normal opening pressure of 16 cm H2O on lumbar puncture. He was started on acetazolamide 250 mg TID. Two weeks post-symptom onset, following a poor-quality outpatient MRI due to dental braces, he was transferred to a tertiary care center; neuro-ophthalmologic evaluation revealed severe vision loss with visual acuity of hand motion OD and counting fingers at face OS and bilateral disc edema on funduscopic exam (Figure 2). He denied pain with eye movement. Brain MRI and orbits demonstrated diffuse abnormal T2 signal and enhancement within the bilateral optic nerves extending into the chiasm, consistent with acute optic neuritis, alongside intraconal fat stranding (Figure 2). Acetazolamide was discontinued. He was treated with IV methylprednisolone (1 g daily for 7 days) with suboptimal visual recovery (20/800 OD and counting fingers at 2 feet OS). He underwent five sessions of PLEX after serum testing positive for MOG-IgG (titer of 1:10,000). CSF MOG antibody levels were not obtained. CSF and serum testing for anti-NMDA receptor antibody were negative. He was started on and maintained on tocilizumab (8 mg/kg every 4 weeks). He was discharged on an oral prednisone taper and was started on tocilizumab maintenance therapy.

**Figure 2:**
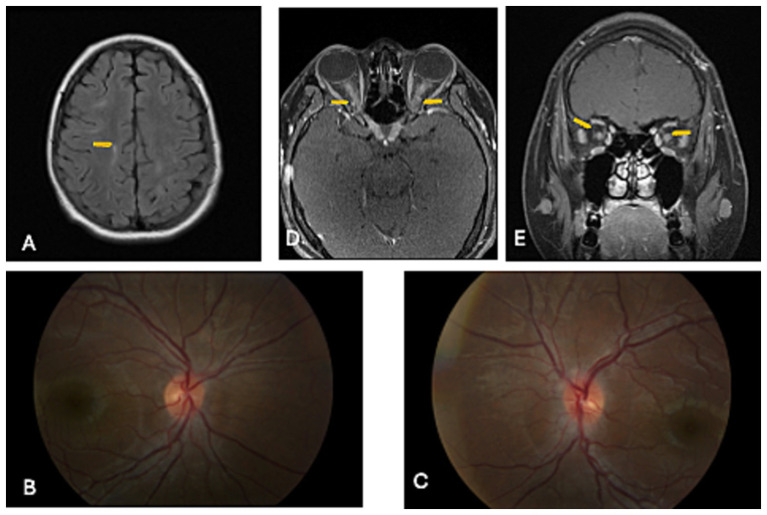
Case 2. **(A)** MRI of the brain shows multiple T2/FLAIR hyperintense lesions in brain parenchyma demonstrated by yellow arrows; **(B, C)** fundus photographs demonstrate bilateral optic nerve edema; **(D)** bilateral optic nerve enhancement on axial T1 post-contrast fat saturated sequence MRI demonstrated by yellow arrows; **(E)** bilateral optic nerve enhancement on coronal T1 post-contrast fat saturated sequence MRI demonstrated by yellow arrows.

At 2 months post-ON onset, examination showed a visual acuity of 20/40-3 OD and 20/25-3 OS, and bilateral optic disc pallor. OCT revealed diffuse GCC thinning OU and superotemporal pRNFL thinning OU. By 4 months, MRI showed resolution of optic nerve enhancement, and visual acuity normalized to 20/20 OU. Five-month neuro-ophthalmology follow-up showed stable visual acuity (20/20-3 OD, 20/25+1 OS), diffuse pallor of both optic nerves, and thinning on OCT (GCC 57/58, RNFL 56/56) with improved visual fields. At 1-year post-presentation, visual acuities were (20/20 OU), and visual fields showed non-specific generalized defects.

### Case 3

A 26-year-old man presented to an outside hospital for evaluation of 1 month of headaches. He was treated for viral meningitis after a lumbar puncture showed an elevated CSF WBC count, along with slightly elevated protein (60 mg/dL) and normal glucose. He was subsequently discharged. After 10 days, he had minimal headache improvement, and he developed altered mental status and photophobia. Optometry evaluation showed bilateral disc edema. He was referred to our ED. Neurologic examination showed decreased attention and concentration, delayed recall, increased deep tendon reflexes, upgoing plantar reflex, and right upper extremity ataxia. Neuro-ophthalmology examination showed visual acuity of 20/25-3 OD and 20/70-2 OS, a left RAPD, and mild disc edema OU. MRI brain with and without contrast revealed multifocal T2 /FLAIR hyperintensities involving bilateral thalami, brainstem, and the optic chiasm (Figure 3). There were increased signal and enhancement in the retrobulbar, intraorbital, canalicular, and prechiasmatic segments of the optic nerves. LP showed a normal opening pressure; CSF studies showed lymphocytosis, elevated protein, and five oligoclonal bands. He was diagnosed with left more than right ON with encephalitis. He was started on IV solumedrol (1 g/day) and subsequently treated with PLEX due to minimal improvement on IV solumedrol. Serum MOG was positive with a 1:10,000 titer. CSF MOG antibody levels were not obtained.

**Figure 3:**
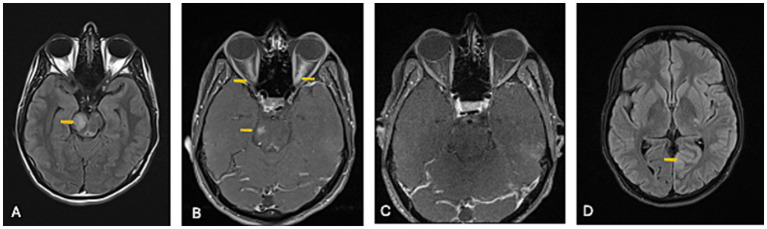
Case 3. **(A)** Axial MRI brain shows T2/FLAIR signal hyperintensity of the bilateral brainstem demonstrated by yellow arrows; **(B)** axial MRI orbits T1 post-contrast fat saturated sequence show enhancement in the bilateral optic nerves demonstrated by yellow arrows; **(C)** resolution of optic nerve enhancement; **(D)** axial MRI brain shows T2/FLAIR hyperintensity within the left medial occipitoparietal lobes demonstrated by yellow arrows.

Following PLEX, his vision improved. Due to ongoing neuro-psychiatric manifestations, a repeat MRI was performed that showed decreased enhancement in the optic nerves and in the multifocal white matter lesions. He was started on rituximab and olanzapine. An encephalopathy/autoimmune panel was positive for NMDAR antibodies in the CSF. He was discharged on rituximab maintenance therapy. Repeat brain MRI 2 months after discharge, while he was on Rituximab, showed an interval increase in white matter lesions; he was switched to IVIG treatment. One month after starting IVIG, he was symptomatically back to baseline. A 9-month follow-up MRI showed radiographic improvement, after which IVIG was tapered and discontinued 2 years after the initial attack in view of clinical and radiographic stability. IVIG was discontinued.

He presented to the ED with a seizure-like episode 5 months after stopping IVIG. The neuro-ophthalmology examination showed 20/20 visual acuity OU with pale optic nerve bilaterally. Repeat MRI brain showed new T2/FLAIR white matter lesions and bilateral optic nerve T2 hyperintensity without enhancement. EEG showed generalized slowing. Repeat LP showed lymphocytosis, NMDAR Ab negative in serum and CSF. He tested positive for serum MOG with a titer of 1:10,000. CSF MOG antibody levels were not obtained. He was diagnosed with a relapse. He improved after treatment with IV steroids and IVIG. He was discharged on an oral steroid taper and maintenance IVIG with continued clinical stability.

## Discussion

Anti-NMDAR encephalitis typically presents with 1–2 weeks of psychiatric and behavioral symptoms (delusions, hallucinations, mania, agitation, disorganization of thinking, changes in speech, catatonia, and insomnia) followed by neurological symptoms (movement abnormalities, seizures, dysautonomia, and hypoventilation) in the following weeks to months. MOGAD optic neuritis typically presents with acute, painful vision loss and severe optic disc edema, characterized by peripapillary hemorrhages and wrinkles, which are present in 70–80% of cases. Bilateral presentation can occur in up to 50% of patients (10). All our patients had headaches, followed by altered mental status during the encephalitis episode. Studies have shown that MNOS patients had higher rates of headache when compared to isolated anti-NMDAR encephalitis (15). All patients developed seizures during their hospitalization. All our patients complained of blurry vision and had either disc edema or disc pallor. These cases highlight that non-specific vision symptoms, such as blurred vision, in a patient with anti-NMDAR encephalitis, may be due to a concurrent demyelinating syndrome. A prompt eye examination is essential to capture acute clinical signs of inflammatory/demyelinating ON, such as anterior optic disc edema, dyschromatopsia, RAPD, or optic nerve pallor, to prevent delayed diagnosis.

A large cohort series showed that MRI was normal in 70% patients (1) with isolated anti-NMDAR encephalitis; optic nerve enhancement is not a typical finding in anti-NMDAR encephalitis. In patients with isolated MOGAD ON, robust enhancement of more than half the optic nerve length occurs in ~80% of patients, with extension into the orbital fat in ~50% (10). However, studies examining the co-occurrence of anti-NMDAR encephalitis and MOGAD have demonstrated higher rates of MRI changes; ~90% have T2/FLAIR enhancing lesions in the cortical and subcortical regions, brainstem, limbic cortex, and/or optic nerves (22). Consistent with those studies, all of our patients had MRI findings during encephalitis; our two sequentially presenting patients had T2/FLAIR hyperintense lesions on MRI during their encephalitis, and our patient with a simultaneous presentation had T2/FLAIR brain lesions, in addition to enhancement of the optic nerves. During the episode of optic neuritis, our two sequentially presenting patients had optic nerve enhancement with interval resolution of their T2/FLAIR hyperintense brain lesions. Therefore, the presence of MRI findings in anti-NMDAR positive patients should raise a high degree of suspicion for dual autoimmunity (MNOS) and may warrant workup for co-occurring demyelinating syndromes.

Serum titers >1:100 for MOGAD antibody, using fixed cell-based assays, are considered significant for MOGAD ON (23). All 3 patients had high MOGAD antibody titers during their ON episodes (1:100, 1:10,000, and 1:10,000). Isolated anti-NMDAR encephalitis is not typically associated with MOG positivity, and dual positivity reflects an overlapping syndrome (MNOS) with findings and symptoms distinct from either individual pathology. CSF positivity is a hallmark of anti-NMDAR encephalitis, and in patients with MNOS syndrome, demonstration of CSF MOG antibody positivity suggests a compartmentalized immune response targeting both oligodendrocytes and neurons. Owing to the retrospective nature of our case review, none of our patients underwent CSF MOG testing.

The therapeutic approach to this dual pathology presents unique clinical challenges: while isolated presentations of MOGAD or anti-NMDAR encephalitis may readily respond to standard first-line agents, the overlapping syndrome frequently dictates a swifter escalation of treatment. A 2021 meta-analysis of 1,550 patients with anti-NMDAR encephalitis from 652 articles reported treatment with IVIG, corticosteroids, therapeutic apheresis, or a combination of these as first-line therapy. Early treatment within 30 days of onset was found to be critical. Delayed treatment resulted in more severe residual disability (24). Rituximab and cyclophosphamide are generally used as second-line agents when the response to first-line agents is limited.

Isolated MOGAD-ON is usually highly responsive to monotherapy with IV steroids. Evidence suggests that early treatment (<2 weeks after onset) not only increased the odds of seronegative conversion but also reduced the risk of relapse (25). Additionally, it has been shown that initiation of high-dose steroids improves visual recovery, with 80–90% showing full visual acuity recovery (final visual acuity 20/20) and better peripapillary RNFL thickness at 3 months (26).

However, data on the treatment of MNOS patients are less clear, and there are limited published data comparing treatment outcomes in MNOS with those in isolated MOGAD-ON or isolated anti-NMDAR encephalitis. A recent systematic review on 256 patients with MNOS suggested that 70% of MNOS patients had a global clinical response to first-line therapy (14). In contrast, a recent cohort study reported that 69% required second-line treatment and long-term immunotherapy to prevent relapse of both anti-NMDAR encephalitis and MOGAD-ON as MNOS patients’ relapses can manifest as either phenotype (16). Additionally, MNOS patients have a higher relapse rate when compared to isolated anti-NMDAR encephalitis.

Thus, dual autoimmunity presents unique therapeutic challenges with regard to the need for escalation of therapy, increased relapses, and the need for long-term maintenance treatment. One patient with sequential anti-NMDAR encephalitis and MOGAD-ON and the patient with simultaneous dual positivity received 5 days of IV steroids followed by 5–7 sessions of PLEX while encephalitic. The other patient with sequential positivity was treated with corticosteroids, followed by IVIG while encephalitic. Rituximab was initiated in all our patients following inadequate therapeutic response. One sequentially positive patient developed MOGAD optic neuritis 4 months after receiving Rituximab. Both sequentially positive patients received 5–7 days of IV methylprednisolone (1 gm/day) during their acute optic neuritis. Both required further acute treatment; one with IVIG and the other with PLEX. Both patients also required long-term maintenance therapy; one was treated with rituximab and the other with tocilizumab. Our patient with simultaneous dual positivity experienced a MOGAD relapse when maintenance therapy was stopped, further necessitating long-term maintenance therapy with IVIG.

## Conclusion

This series highlights the complex interplay between anti-NMDAR encephalitis and MOGAD-associated ON. ON may occur simultaneously or sequentially with the encephalitic presentation, underscoring the need for careful monitoring of patients with anti-NMDAR encephalitis and new visual symptoms. Dual autoimmunity may represent a distinct phenotype with alterations in the clinical course, imaging profile, and treatment considerations compared to either disease alone. There is a need for larger prospective studies to better understand the diagnostic, therapeutic, and prognostic implications of this dual autoimmune condition. Clinicians should adopt a low threshold for MOG antibody testing in NMDAR patients with vision loss.

## Data Availability

The datasets presented in this article are not readily available because of ethical and privacy restrictions. Requests to access the datasets should be directed to the corresponding author.
