# Supplementary material for: Anti-NMDA-receptor encephalitis and MOGAD associated optic neuritis: a case series
Source: Front Neurol. 2026 Jul 10;17:1869098. doi: 10.3389/fneur.2026.1869098 (PMC13395603; doi:10.3389/fneur.2026.1869098)
Supplement: Supplementary file 1 [file Table_1.DOCX]

| **Sl.No** | **Age/Gender** | **Initial presentation** | **Clinical signs** | **MRI brain with and without contrast** | **Antibody testing** | **Treatment** | **Interval presentation** | **On examination** | **MRI brain and orbits with and without contrast** | **Antibody testing** | **Treatment** |
| --- | --- | --- | --- | --- | --- | --- | --- | --- | --- | --- | --- |
| 1 | 36/F | Headache,altered mental status | Encephalopathic, aphasic with Normal ophthalmology exam | Abnormal increased T2/FLAIR signal and associated gyriform edema predominantly involving the left frontal, temporal, parietal lobes. Additional evidence of multifocal punctate enhancing lesions involving the pons, cerebellum, and medulla as well as scattered areas of leptomeningeal enhancement with involvement of the cranial nerves. | CSF Anti NMDAR Ab positive 1:256 | IV steroids, PLEX, rituximab | Blurry vision of the left eye 4 months after initial presentation | Reduced visual acuity in the left eye, severe dyschromatopsia, left RAPD. Temporal pallor of left eye | Interval resolution of leptomeningitis and encephalitis and perineural enhancement of left> right optic nerves | Serum Anti MOG antibody positive 1:100 | IV steroids and IVIG f/b Rituximab maintainence |
| 2 | 9/M | Headache, altered mental status | Encephalopathic with response to painful stimulation | Abnormal increased T2/FLAIR hyperintensities in multiple locations in the bilateral parenchyma | CSF Anti NMDAR Ab positive | IV steroids, IVIG, Rituximab | Right>left blurry vision 10 years later | Bilateral asymmetrically reduced visual acuity and dyschromatopsia. Bilateral disc edeme on funduscopic exam | Diffuse abnormal increased T2 signal within the optic nerves bilaterally with associated enhancement and extension into the optic chiasm. There is surrounding fat stranding within the intraconal fat bilaterally. | Serum MOG positive 1:10000 | IV steroids, IVIG, tocilizumab |
| 3 | 26/M | Headache, altered mental status and bilateral blurry vision | Disoriented with manic episode.Bilateral asymmetrically reduced visual acuity and reduced color plates. Bilateral disc edema on funduscopic exam, | Multifocal areas of FLAIR intensity involving the bilateral thalami, hypothalamus, right midbrain, medulla, and the craniocervical junction/apico-cervical cord at C1. Increased T2 signal and enhancement of bilateral optic nerves, optic chiasm, and optic tracts | CSF Anti NMDAR Ab positive  Serum MOG positive 1:1000 | IV steroids, PLEX, IVIG |  |  |  |  |  |

Table 1: Description of the cases. F- Female, M- Male, CSF – cerebrospinal fluid, NMDAR- N-methyl-D-Aspartate receptor, Ab- Antibody, PLEX- Therapeutic plasma exchange, MOG- myelin oligodendrocyte glycoprotein.
